# Host-Microbiome Integration as a Biomarker Framework in Esophageal Cancer: Current Evidence and Translational Challenges

**DOI:** 10.3390/cimb48080831

**Published:** 2026-08-16

**Authors:** Shamimeh Pourbahrighesmat, Alireza Tojjari, George Laliotis, Anwaar Saeed

**Affiliations:** 1 Department of Medicine, Division of Hematology & Medical Oncology, University of Pittsburgh Medical Center (UPMC), Pittsburgh, PA 15213, USA; pourbahrighesmats@upmc.edu (S.P.); tojjaria@upmc.edu (A.T.); laliotisg@upmc.edu (G.L.); 2 UPMC Hillman Cancer Center, 5150 Centre Avenue, Pittsburgh, PA 15213, USA

**Keywords:** esophageal cancer, immunotherapy, microbiome, immune checkpoint inhibitors, biomarkers, tumor microenvironment, microbial metabolites, multi-omics

## Abstract

Immune checkpoint inhibitors have improved outcomes in esophageal cancer across settings, yet clinical benefit remains heterogeneous, with current host-derived biomarkers incompletely predicting response. This mini review evaluates recent studies that integrate gut or intratumoral microbial features with host immune, molecular, or metabolic assessment in esophageal cancer. We classify the evidence using a four-level hierarchy of host-microbiome integration: ecological association, functional association, mechanistic integration, and clinical predictive integration. Tissue studies reveal compartment-specific relationships between microbial diversity or individual taxa and immune architecture, whereas treatment cohorts identify bacterial and fungal signatures associated with pathological or immunotherapy response. Mechanistic studies offer the strongest biological evidence, most notably the *Lactobacillus salivarius*-indole-3-lactic acid-AhR/NF-κB axis, which drives CD8-positive T-cell exhaustion and resistance to anti-PD-1 therapy. However, biological integration is substantially more advanced than clinical response prediction. Small cohorts, heterogeneous regimens, contamination of low-biomass samples, coarse taxonomic (rather than functional) resolution, confounding by histology, multi-omic layers measured in different patients, and lack of external validation currently jeopardize integration of microbiome to guide treatment. Future studies should use longitudinal, multicenter, compartment-matched sampling and test whether microbial genes or metabolites improve patient selection and predict clinical response beyond established clinical and host biomarkers.

## 1. Introduction

Immunotherapy has substantially transformed the treatment landscape of esophageal cancer, in both esophageal squamous cell carcinoma and adenocarcinoma [1,2,3,4]. Immune checkpoint inhibitors are now used in multiple treatment settings, and a subset of patients achieves profound and durable responses [3,5,6,7]. However, therapeutic benefit remains highly heterogeneous: while some patients experience dramatic tumor regression, others derive little or no benefit despite receiving similar treatment regimens [3,5]. This variability has created an urgent need for reliable biomarkers to identify patients most likely to respond.

Several host-derived biomarkers, including programmed death-ligand 1 (PD-L1) expression, microsatellite instability or mismatch-repair deficiency (dMMR), tumor mutational burden (TMB), immune-cell infiltration, and tumor transcriptional signatures, have been investigated as predictors of treatment response [8,9]. Although these biomarkers capture important aspects of tumor immunogenicity and host antitumor immunity, none alone can adequately explain or predict the full spectrum of clinical responses [10]. Their performance may also be limited by spatial and temporal heterogeneity, differences in assay methodology, and the complex interaction between tumor-intrinsic features and the surrounding immune microenvironment [11].

Recent studies have therefore begun to incorporate the host-associated microbiome into biomarker research [12,13,14]. Gut, oral, and intratumoral microorganisms may influence immunotherapy efficacy through microbial metabolites, inflammatory signaling, immune-cell recruitment, and regulation of T-cell function [15,16,17]. Emerging investigations in esophageal cancer have combined microbial profiles with one or more of the host immune-cell phenotypes, transcriptomics, epigenomics, metabolomics, and conventional immune biomarkers [16,17,18,19,20,21,22,23,24]. However, the degree of integration, biological interpretation, and predictive value varies substantially across studies.

In this mini review, we evaluate studies that examined microbial features together with host immune, molecular, or metabolic biomarkers in esophageal cancer. We assess how these data layers were integrated, their contribution to treatment-response prediction or mechanistic interpretation, and which methodological limitations currently prevent clinical translation. Rather than cataloging microbial taxa, this mini review critically evaluates the depth of host-microbiome integration and its clinical utility. We further propose practical strategies for developing reproducible and clinically informative host-microbiome biomarker models for personalized treatment selection.

In this review, we define host-microbiome integration as the extent to which microbial features are linked to host phenotypes, molecular processes, immune function, or clinical prediction within a shared analytical or experimental framework. To distinguish biologically and clinically distinct forms of integration, we classify the evidence using a four-level evidentiary-translational hierarchy. Level 1, ecological association, encompasses associations between microbial composition, diversity, or individual taxa and host immune phenotypes, prognosis, or treatment outcomes, without evidence of functional mediation or causality. Level 2, functional association, extends beyond microbial identity to microbial genes, pathways, or metabolites linked to host molecular or immune biology, but without a fully demonstrated causal chain. Level 3, mechanistic integration, requires experimental evidence linking a defined microbial factor to a specific host molecular pathway and a downstream immune or treatment-related consequence. Level 4, clinical predictive integration, requires a validated model demonstrating that microbial features provide incremental predictive value beyond a prespecified benchmark incorporating established clinical and host biomarkers. These levels reflect increasing inferential and translational depth, and studies are classified according to the highest level supported by their data. Importantly, mechanistic evidence does not itself establish clinical predictive utility, and an association between a microbial feature and treatment outcome should not be considered evidence of treatment-specific prediction without appropriate predictive validation.

### Literature Search Strategy

A focused narrative search of PubMed/MEDLINE, Scopus, and Web of Science was conducted from database inception through 29 June 2026. Search terms combined “esophageal cancer,” “esophageal squamous cell carcinoma,” or “esophageal adenocarcinoma” with “microbiome,” “microbiota,” “bacteria,” or “fungi,” and terms related to host biomarkers, multi-omics, immunity, treatment response, and prognosis. Original English-language human and translational studies linking microbial features with host molecular, immune, or metabolic biomarkers were included. Studies limited to descriptive microbial taxonomy or associations with clinical outcomes without measurement of a corresponding host immune, molecular, or metabolic feature were excluded, as were studies in which host variables were used solely for subgroup annotation rather than for host-microbiome integration.

## 2. Current Evidence for Host-Microbiome Integration

The following sections organize the reviewed studies according to the highest level of host-microbiome integration supported by their data.

### 2.1. Ecological Association

#### 2.1.1. Microbial Composition Associated with Host Immune Phenotype and Prognosis

The earliest integration strategy measured microbial abundance in resected tissue and related it to immune phenotypes in the same tumor. Across these studies, no single organism marked a uniformly “favorable” or “unfavorable” immune state. In 98 resected esophageal squamous cell carcinomas (ESCC), greater intratumoral bacterial diversity and higher *Lactobacillus* abundance were associated with poorer overall survival. Greater diversity accompanied fewer natural killer cells and more PD-L1-positive epithelial cells and tumor-associated macrophages, while *Lactobacillus* abundance was also positively related to PD-L1-positive epithelial and macrophage compartments [18]. This pattern is consistent with an immunosuppressive local ecology, but the cross-sectional design cannot distinguish microbial induction of immune suppression from preferential colonization of an already permissive tumor.

Single-taxon studies emphasized the anatomical picture but also demonstrated why immune associations should not be generalized. In 300 resected esophageal cancers, intratumoral *Fusobacterium nucleatum* (*F. nucleatum*) was independently associated with weaker peritumoral lymphocytic reaction, yet not with CD8-positive, FOXP3-positive, or PDCD1-positive cell densities, other lymphocytic-reaction patterns, or CD274, PDCD1LG2, and IDO1 expression [19]. Thus, its principal host correlate was immune organization at the invasive margin rather than global T-cell density. By contrast, Bifidobacterium positivity in 213 ESCCs was associated with lower FOXP3-positive cell density in tumor and adjacent tissue, but not with CD8, PD-1, PD-L1, or overall survival; tumor positivity was also associated with a lower global tumor-infiltrating lymphocyte (TIL) score [20]. The combination of reduced regulatory T cells without increased cytotoxic infiltration is therefore not sufficient to characterize *Bifidobacterium* immunostimulatory.

These tissue studies collectively indicate that microbial-host associations are compartment- and phenotype-specific. They also expose a second source of confounding: systemic host condition. Peritumoral *Bifidobacterium* was associated with greater skeletal-muscle area and volume, whereas tumor positivity was associated with lower albumin and prognostic nutritional index [20]. Nutrition and body composition may shape colonization, immune competence, and outcome simultaneously. Consequently, taxon-immune correlations should be interpreted as ecological associations, not causal effects, until directionality is tested experimentally.

#### 2.1.2. Microbial Composition Associated with Treatment Outcome

These treatment cohorts asked a different question from the resection cohorts above: not what the microbiome reveals about prognosis, but whether pretreatment microbial features are associated with treatment response and may support future prediction. However, high microbial diversity is not inherently good or bad; whether it signals a better or worse outcome depends on where and when the sample was taken. Although high intratumoral bacterial diversity predicted poor surgical outcome [18], higher fecal bacterial diversity and higher gut fungal diversity characterized immunotherapy responders [21,22]. This is not necessarily contradictory: tumor diversity may reflect local barrier disruption and an immunosuppressive niche, while fecal diversity may indicate a resilient systemic ecosystem. It does mean that “high diversity” cannot be treated as a universal biomarker without specifying compartment, microbial kingdom, treatment, and time point.

At the tumor level, 25 patients receiving neoadjuvant chemoimmunotherapy showed response-associated differences in beta diversity, with *Streptococcus* enriched in responders. Its abundance correlated with CD8-positive and granzyme B-positive infiltration, along with superior disease-free survival [23]. Fecal microbiota or colonization with *Streptococcus* coming from clinical responders, increased intratumoral CD8-positive cells and anti-PD-1 activity in mice, while CD8 depletion removed this benefit [23]. These experiments move the observation beyond correlation, although the human cohort and single-cell subset were small and no external patient-level classifier combined microbial and immune features.

An oral-compartment study extended these observations to chemoradiotherapy. In initially inoperable LAESCC, baseline salivary microbial features were associated with pathological complete response to chemoradiotherapy and treatment-related immune changes. However, the small cohorts and lack of an externally validated integrated model make these findings exploratory [24].

Stool-based studies broadened the signal to bacteria, fungi, and different histologies. In a predominantly adenocarcinoma cohort of 23 operable esophageal or gastroesophageal-junction cancers treated with nivolumab-based therapy plus chemoradiation, pathological complete response was associated with a distinct fecal community and enrichment of *Ruminococcus callidus*, *Fusicatenibacter saccharivorans*, and *Roseburia inulinivorans* [25]. This is the main evidence among the included studies extending beyond ESCC, but it remains exploratory because only eight patients achieved complete response. In ESCC, a fungal analysis of 105 patients identified higher pretreatment diversity and responder-enriched fungi associated with Th1 cytokines, whereas nonresponder taxa correlated with IL-4, IL-10, and IL-13 [21]. Fungal-only classifiers retained discrimination in an independent test cohort, with AUCs of 0.829 at genus level and 0.874 at species level [21]. However, the cytokines explained the fungal signal but were not included as coequal predictors, so this was biological integration rather than a joint host-mycobiome model.

The most treatment-specific taxonomic signal was fecal *Lactobacillus salivarius*, which was enriched among nonresponders to neoadjuvant anti-PD-1 therapy in a 43-patient discovery cohort and two validation cohorts [22]. Its lack of association with response or survival in a separate chemoradiotherapy cohort supports treatment specificity but does not establish formal treatment-predictive value without a treatment comparator or interaction analysis [22]. Together, these studies demonstrate that response-associated taxa are not interchangeable: intratumoral *Streptococcus* and selected gut fungi accompanied immune activation, whereas gut *L. salivarius* marked a defined resistance pathway [21,22,23].

#### 2.1.3. Microbiome Associations with Immune-Related Adverse Events

Beyond treatment efficacy, the gut microbiome may also be associated with immune-related adverse events (irAEs) during immune checkpoint blockade. In a prospective metagenomic study of 95 patients with gastrointestinal cancers receiving immune checkpoint inhibitors, including 27 patients with esophageal cancer, baseline gut microbial alpha and beta diversity did not differ significantly according to irAE occurrence or severity within the esophageal-cancer subgroup [26]. However, species-level and functional differences were observed. *Ruminococcus torques* was enriched in patients without irAEs, whereas *Dialister invisus* and *Eubacterium ventriosum* were enriched in patients with high-grade compared with low-grade irAEs. At the functional level, microbial pathways related to ubiquinol-6 and glutamine biosynthesis were also enriched in the high-grade irAE group [26]. These findings suggest that specific microbial taxa and functional pathways, rather than overall community diversity, may be associated with immunotherapy toxicity in esophageal cancer. However, the esophageal subgroup was small, the findings were exploratory, and neither external validation nor experimental confirmation of the implicated pathways was performed. Accordingly, these data should be regarded as preliminary functional associations rather than validated biomarkers of irAE risk.

### 2.2. Functional Association

Taxonomy becomes more biologically informative when linked to microbial function, but not every taxon-metabolite correlation establishes a host pathway. In the predominantly adenocarcinoma study, fecal C16 ceramide and chenodeoxycholic acid were enriched in complete responders and correlated most strongly with *Ruminococcus Callidus* [25]. These findings connect community structure to metabolic output, yet both metabolites may reflect microbial production, host metabolism, diet, or co-metabolism. Because no immune, genomic, or transcriptomic host endpoint was measured, this study supports microbiome-metabolome association with pathological response, not direct host-microbiome integration.

More mechanistic work has investigated microbe-metabolite-host interactions in ESCC. Multi-omic tissue profiling linked *F. nucleatum* and *Fusobacterium* abundance to inflammatory transcriptional pathways, tumor-associated macrophages, and increased CLEC12A expression [27]. Phenyllactic acid emerged as a candidate *F. nucleatum*-associated metabolite that increased CLEC12A in THP-1 macrophages and favored M2-associated markers. CLEC12A knockdown reduced *F. nucleatum*-induced M2 polarization and IL-4/IL-10 production [27]. The study therefore connects bacterium, metabolite, host receptor, and macrophage profiling. Nevertheless, the reported survival difference for *F. nucleatum* was not statistically significant. Therefore, it is best viewed as pathway discovery rather than clinical prediction.

Fungal findings occupy an intermediate position. Responder-associated *Candida boidinii* enhanced anti-PD-1 activity, CD8^+^ infiltration, and favorable metabolic programs in a syngeneic model [21]. This validates biological activity but does not yet identify a single fungal metabolite-receptor pathway comparable to the *L. salivarius* axis. Collectively, the metabolite studies suggest that functional pathways may generalize better than taxonomic abundance, but only when microbial origin, host target, and immune consequence are experimentally separated.

### 2.3. Mechanistic Integration

Additional mechanistic evidence links intratumoral *Fusobacterium nucleatum* to immune suppression and resistance to PD-1 blockade in ESCC. Intratumoral *F. nucleatum* DNA and serum anti-*F. nucleatum* IgG levels were higher among immunotherapy nonresponders, while bacterial abundance correlated positively with tumor PD-L1 protein expression. Mechanistically, the bacterial virulence factor *F. nucleatum* DPs (Fn-Dps), which is a secreted ferritin-like virulence protein involved in bacterial oxidative-stress tolerance and intracellular survival, entered the nuclei of ESCC cells and promoted ATF3-dependent transcriptional upregulation of PD-L1 and impaired T-cell proliferation and cytokine secretion. These findings directly connect a microbial feature with host humoral, tumor, and immune-functional markers, although their predictive value requires prospective external validation [28].

The *L. salivarius*-indole-3-lactic acid axis is probably the most complete causal chain reported. Longitudinal human profiling linked *L. salivarius*, tryptophan metabolism, and elevated indole-3-lactic acid to anti-PD-1 nonresponse [22]. In orthotopic ESCC models, an indole-3-lactate dehydrogenase-deficient bacterial strain lost both metabolite production and the ability to induce resistance; purified indole-3-lactic acid restored the phenotype. Single-cell and functional studies showed that the metabolite activated aryl hydrocarbon receptor (AhR), suppressed NF-κB signaling, and promoted loss and terminal exhaustion of NKG7-positive CD8-positive progenitor-exhausted T cells. AhR deletion or pharmacological NF-κB activation restored T-cell function [22]. The specificity of the effect for anti-PD-1 rather than cisplatin or conventional chemoradiotherapy further strengthens causality.

#### Framework for Causality Assessment

Distinguishing mechanistic integration from association requires evidence that moves beyond co-occurrence toward experimentally supported causal relationships. Several complementary approaches can strengthen causal inference in host-microbiome studies. Longitudinal patient sampling can help establish temporality by determining whether microbial changes precede alterations in host immune states or treatment outcomes. Microbial genetic manipulation can identify specific microbial genes or functions required for an observed host effect, while rescue experiments can provide stronger evidence of mediation by restoring the phenotype after reintroduction of the implicated microbial product. On the host side, genetic or pharmacological perturbation of candidate receptors and signaling pathways can establish whether the microbial effect depends on a defined molecular axis. Gnotobiotic, controlled-colonization, or controlled-infection models can directly test microbial effects under experimentally manipulated conditions, whereas patient-derived cells, organoids, and co-culture systems can assess whether candidate mechanisms are retained in a human-relevant biological context [29].

No single experimental approach is sufficient to establish causality across the complex host-microbiome interface; rather, causal confidence increases when multiple complementary lines of evidence converge [29]. This distinction is illustrated by the strongest mechanistic studies identified in this review. Zhou et al. combined longitudinal patient sampling with controlled microbial exposure, targeted deletion of the *L. salivarius ildh* gene, rescue with exogenous ILA, AhR disruption and NF-κB pathway perturbation, and experiments using patient-derived ESCC and immune cells, collectively supporting the *L. salivarius-ILA-AhR/NF-κB* axis [22]. Li et al. similarly moved beyond clinical association by experimentally demonstrating that *Fn* attenuates checkpoint-blockade efficacy and that Fn-Dps induces PD-L1 through ATF3-dependent regulation [28]. These convergent perturbational data justify classification as mechanistic integration, whereas studies demonstrating microbial-host correlations or functional associations without experimental interrogation of the causal chain remain at lower levels of the hierarchy.

### 2.4. Clinical Predictive Integration: Incremental Value Beyond Established Biomarkers

Clinical predictive integration represents the highest level of the proposed hierarchy and requires more than an association between microbial features and treatment outcome. To meet this level, microbial variables should provide demonstrable incremental predictive value beyond a prespecified model containing established clinical and host biomarkers, with performance confirmed in an independent cohort using a locked analytical pipeline. Under this definition, none of the studies reviewed here fully meets Level 4. The integrated model reported by Zhou et al. [22] represents the closest candidate, but it has not demonstrated incremental performance over an established clinical-plus-host benchmark or undergone locked external validation.

Analyzing multi-omics layers does not automatically produce valid clinical integration. A TCGA-based study combined bacterial abundance, RNA expression, DNA methylation, mutations, immune deconvolution, and clinical data to define two esophageal-cancer subtypes [30]. The subtype with greater *Pseudomonas* abundance also showed higher tumor mutational burden, immune and stromal scores, regulatory T cells, M2 macrophages, but lower TIDE (Tumor Immune Dysfunction and Exclusion) scores and a higher computationally predicted response rate [30]. Yet all tumors in that subtype were adenocarcinomas, whereas 86% of the other subtype were squamous carcinomas. Histology can therefore explain much of the molecular, immune, prognostic, and microbial separation. Moreover, treatment response was primarily computationally inferred, and experimental validation tested subtype-specific host pathways rather than microbial causality. This study illustrates that unsupervised multi-omics can amplify confounding unless histology and other dominant covariates are explicitly controlled.

The *L. salivarius* study is the only study reviewed here that adopted a comprehensive clinical model. This model integrated bacterial abundance with the expression of PD-L1 and the proportion of NKG7^+^ CD8^+^ progenitor-exhausted T cells [22]. In the discovery cohort, baseline *L. salivarius* abundance discriminated responders from non-responders better than PD-L1 expression [22]. The study therefore provides strong mechanistic integration and a promising candidate for clinical prediction. However, validation of the individual microbial, metabolic, and immune signals should not be equated with validation of a complete host-microbiome predictive model. Incremental performance beyond a prespecified clinical-plus-host benchmark and locked external validation of the integrated model remain to be demonstrated. Table 1 summarizes these studies and their key findings.

## 3. Discussion

Taken together, the reviewed studies describe different levels of host-microbiome integration rather than a set of clinically equivalent biomarkers. Within this review, host-microbiome integration refers to the extent to which microbial measurements are connected to host biology and clinical prediction. The evidence can be organized into four levels: ecological association, in which microbial composition is associated with immune phenotype, prognosis, or treatment outcome; functional association, in which microbial genes, pathways, or metabolites are linked to host biology; mechanistic integration, in which a defined microbial factor acts through a host molecular pathway to produce an immune consequence; and clinical predictive integration, in which an externally validated model demonstrates improvement beyond established clinical and host biomarkers. Most studies reviewed here support ecological or functional associations [18,19,20,21,23,24,25,27,30], while only a few provide mechanistic evidence [22,28]. No study has yet established clinically validated predictive integration. Table 1 therefore classifies each study according to the highest level directly supported by its design. This hierarchy reinforces the central conclusion of the review: biological understanding is currently more advanced than clinical prediction.

These distinctions also support a transition from microbial identity to microbial function as the next generation of biomarkers. Taxonomic abundance alone may not generalize across studies because organisms classified within the same genus or species can differ in strain-specific genes, transcriptional activity, metabolite production, and interactions with other members of the microbial community. This may help explain why tumor-associated *Lactobacillus* abundance was linked to an immunosuppressive phenotype and poorer prognosis, whereas a specific fecal *L. salivarius* strain was associated with anti-PD-1 resistance through defined tryptophan metabolism [18,22]. Future studies should therefore integrate strain-level metagenomics, microbial gene expression, targeted metabolomics, pathway activity, and ecological interactions. The *L. salivarius*-indole-3-lactic acid pathway illustrates the value of this functional approach: disruption of the bacterial gene required for metabolite production removed the resistance phenotype, while metabolite rescue restored it [22]. Nevertheless, inferred pathways and taxon-metabolite correlations should not be considered demonstrated microbial function without direct measurement and experimental perturbation [29].

Histology is another major determinant of interpretation. ESCC and esophageal adenocarcinoma differ in anatomical origin, environmental exposures, molecular characteristics, immune context, microbial niche, and treatment strategies [1,2,3,5,6]. ESCC develops from native squamous epithelium and is strongly associated with exposures such as tobacco and alcohol, whereas adenocarcinoma typically arises in the distal esophagus in the context of reflux and Barrett-associated columnar transformation. These differences are likely to expose the tumors to distinct microbial environments, although direct comparative host-microbiome studies remain limited. Importantly, most of the mechanistic evidence reviewed here was generated in ESCC [22,27,28]. Evidence relevant to adenocarcinoma is largely limited to a small, predominantly adenocarcinoma cohort showing associations between fecal taxa, metabolites, and pathological response [25]. Furthermore, the TCGA-based multi-omic study was strongly confounded by histology, because its microbiome-associated subtypes largely separated adenocarcinoma from squamous carcinoma [30]. Future studies should therefore prespecify histology-stratified analyses and should not assume that biomarkers developed in ESCC are applicable to adenocarcinoma.

A structured framework is also needed to distinguish mechanistic evidence from correlation. Confidence in causality should increase when a study demonstrates: longitudinal temporal ordering; strain-level identification and microbial genetic manipulation; depletion and rescue of the proposed metabolite or microbial product; validation of the relevant host receptor or molecular pathway; reproduction of the immune phenotype in gnotobiotic or controlled-colonization models; and confirmation in patient-derived experimental systems. The *L. salivarius* study provides the most complete causal chain because it combines longitudinal human observations, microbial gene disruption, metabolite rescue, host-pathway perturbation, and treatment-specific in vivo effects [22]. Responder-derived *Streptococcus* colonization and loss of benefit following CD8 depletion provide functional evidence but do not identify a specific microbial product and host receptor [23]. Similarly, CLEC12A knockdown and Fn-Dps-mediated ATF3 activation strengthen the proposed *F. nucleatum* pathways, although their clinical predictive value remains unvalidated [26,27].

The oral microbiome represents an especially relevant but underdeveloped compartment. Because the oral cavity and esophagus form a continuous mucosal ecosystem, saliva may provide an accessible source of biomarkers and permit repeated sampling during therapy. In LAESCC, baseline salivary microbial and metabolic features were associated with pathological response to chemoradiotherapy and accompanied treatment-related immune changes [24]. Prospective studies have also examined oral microbial composition in relation to esophageal-cancer risk, although findings have not been uniformly reproducible [31]. Salivary composition should not be assumed to represent the tumor microbiome because it is influenced by oral health, smoking, alcohol exposure, antibiotics, diet, and oral-hygiene practices. Future studies should collect matched saliva, esophageal tissue, stool, and blood and use strain-resolved source tracking to test microbial migration and ecological transition.

**Figure 1:**
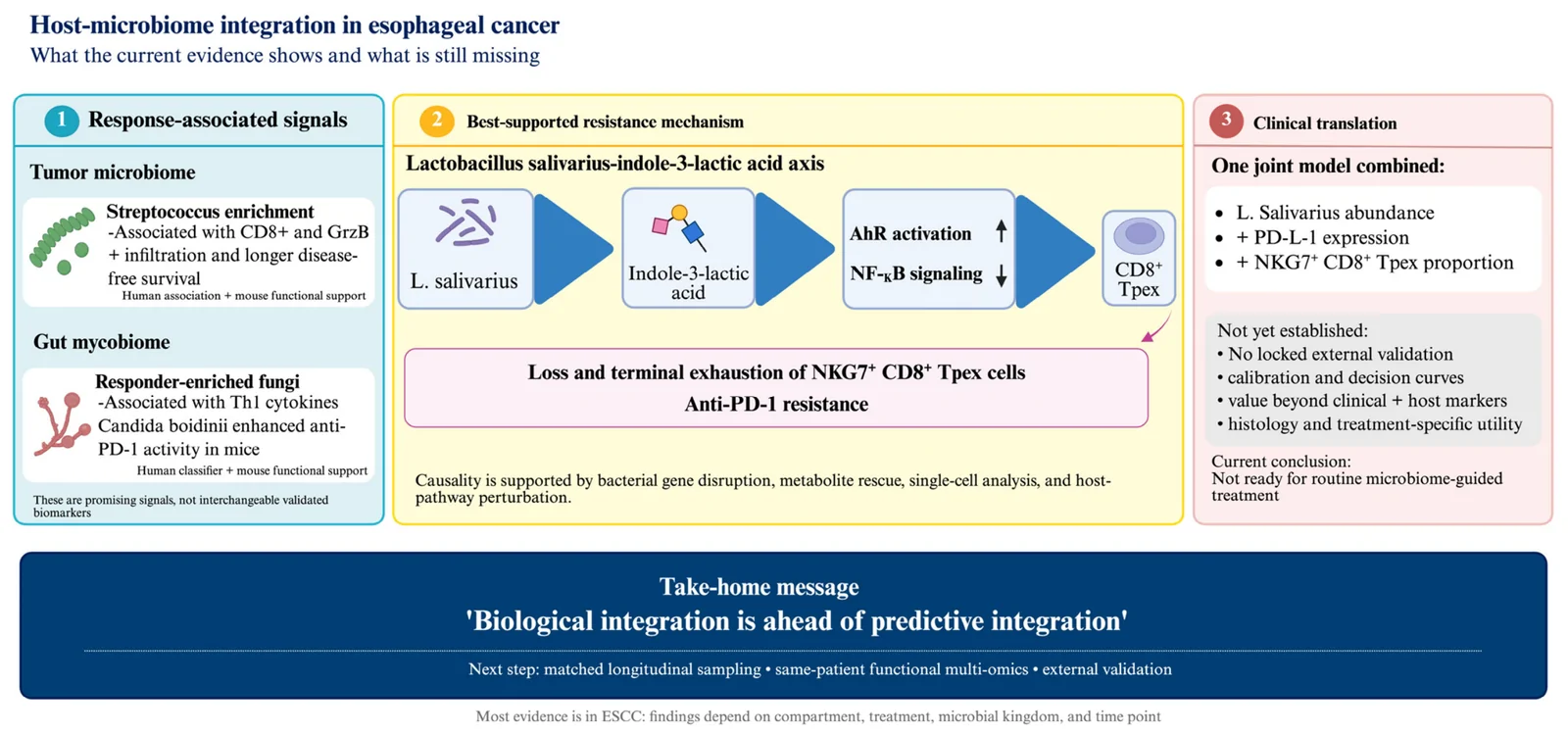
Current state of host-microbiome integration in esophageal cancer. Response-associated microbial signals and mechanistic pathways have been identified, but integrated clinical prediction remains limited by insufficient external validation and uncertain incremental value beyond established clinical and host biomarkers. Created in BioRender. Tojjari, A. (2026) https://BioRender.com/4tgdbeo.

Clinical translation will ultimately depend on analytical reproducibility and demonstrated incremental predictive value. Differences in specimen collection, storage, DNA extraction, sequencing platform, bioinformatic processing, antibiotic exposure, diet, and geographic population can substantially affect microbial measurements [32,33]. Multicenter studies should therefore use standardized procedures, positive and negative controls, contamination-aware processing, and predefined assay thresholds. Machine-learning approaches may facilitate multi-omic integration but are particularly vulnerable to overfitting, feature-selection instability, data leakage, and limited interpretability in small cohorts. Internal cross-validation cannot replace locked external evaluation. Future models should report discrimination, calibration, and decision-curve analysis and should demonstrate improvement beyond a prespecified clinical-plus-host benchmark in independent populations, consistent with TRIPOD+AI guidance [34]. Until these requirements are met, microbiome findings should be regarded as biologically informative and hypothesis-generating rather than ready for treatment selection. Representative response-associated microbial signals, the best-supported mechanistic pathway, and the remaining gap in clinical prediction are summarized in Figure 1.

## 4. Current Limitations and Translational Challenges

Tissue-based observational methods are limited mainly by directionality, spatial resolution, and low microbial biomass. Cross-sectional analysis of resected specimens cannot determine whether microbes reshape the immune microenvironment or preferentially colonize tumors that are already inflamed or immunosuppressed. Bulk tissue also combines epithelial, stromal, invasive-margin, and immune-cell-associated signals, although microbial effects may be restricted to one niche. Archived or low-biomass specimens are vulnerable to reagent and environmental contamination, while adjacent mucosa may already be altered by field effects or treatment. These limitations require longitudinal sampling, rigorous negative controls, orthogonal localization, and spatially resolved host and microbial measurements [18,19,20,30].

Pre-analytical and analytical reproducibility must therefore be established before any microbial signature is interpreted as biological or predictive. Studies should standardize and report the biopsy collection site and timing, the interval from collection to processing, and tissue-handling procedures, as well as specimen state (fresh or frozen), storage temperature, time to freezing, stabilization method, and freeze–thaw exposure. DNA-extraction protocols and kits can preferentially recover or lose specific taxa, while 16S rRNA gene sequencing, ITS sequencing, shotgun metagenomics, sequencing platform, target region, library preparation, and read depth provide different taxonomic and functional resolution. Because esophageal tissue is typically low biomass, extraction blanks, reagent and environmental controls, positive controls, and orthogonal confirmation are essential to distinguish genuine signals from contamination. Responders and nonresponders should be randomized or balanced across extraction and sequencing batches, with batch variables recorded and assessed to prevent technical differences from mimicking biology. Bioinformatic choices (including quality filtering, contaminant removal, reference database, taxonomic assignment, normalization, and handling of low-abundance features) should be prespecified and validated. Cross-laboratory replication using common reference materials is needed to demonstrate that a candidate signature is not an artifact of collection, extraction, sequencing, or computational processing.

Taxonomic profiling methods provide insufficient functional resolution. The use of 16S sequencing, ITS2 sequencing, or genus-specific qPCR frequently limits interpretation to genus or approximate species level and cannot establish strain-specific activity, microbial viability, or active gene expression. Predicted functional pathways cannot substitute for shotgun metagenomics, metatranscriptomics, culture, or direct metabolomics. A taxon may also serve only as a proxy for an unmeasured community function, making results difficult to reproduce across populations with different strains or ecological backgrounds [18,19,20,21,23,25,27].

### 4.1. Ecological and Clinical Confounding

Even when microbial measurement is analytically reliable, ecological and clinical confounding can generate or distort associations with treatment response. Antibiotic and other medication exposure should be recorded relative to each sampling time point; diet, geographic setting, oral health and hygiene, and nutritional status and body composition may reshape microbial communities and also correlate independently with treatment tolerance or outcome. Histology and treatment regimen are especially important: esophageal squamous cell carcinoma and adenocarcinoma differ in anatomy, exposures, microbial ecology, and treatment patterns, while disease setting and co-administered chemotherapy or chemoradiotherapy may influence both microbial profiles and response. Sampling compartments (including tumor, adjacent mucosa, saliva, and stool) are not interchangeable, and sampling time before treatment, during therapy, after antibiotic or nutritional interventions, or at progression can determine whether a signature reflects baseline biology, treatment-induced change, or disease evolution. Study design and multivariable analysis should therefore prespecify these variables, use compartment- and time-matched sampling, and assess whether associations persist within histology and treatment strata.

Treatment-response cohort methods are constrained by small samples, heterogeneous regimens, and inadequate comparator groups. Limited numbers of responders increase the risk of unstable taxa and exaggerated predictive performance, especially when feature selection and model evaluation occur in the same cohort. Combining different immunotherapy, chemotherapy, or chemoradiotherapy regimens can obscure whether a microbial feature predicts checkpoint-blockade benefit or merely reflects general treatment sensitivity. Without a parallel non-immunotherapy cohort or formal treatment-interaction analysis, prognostic and treatment-predictive effects cannot be reliably separated [21,22,23,25].

Multi-omic integration introduces additional problems when molecular layers are not measured in the same patients or when dominant clinical variables are insufficiently controlled. Correlations across separate microbiome, transcriptome, and metabolome subsets may reflect cohort differences rather than within-patient biological coupling. Untargeted metabolites may originate from microbes, the host, diet, or co-metabolism, so metabolite origin requires isotope tracing, culture, or microbial genetic perturbation. Unsupervised clustering can also reproduce histological differences rather than discover microbiome-defined biology, and computationally predicted treatment response is not equivalent to observed response [25,27,30].

Experimental methods improve causal inference but do not automatically ensure human translation. Cell lines and mouse models simplify immune-cell diversity, microbial colonization, exposure duration, and metabolite concentrations. A convincing causal chain requires manipulation of the microbial gene or metabolite, rescue of the phenotype, perturbation of the proposed host receptor, and confirmation in patient-derived systems. Even when these conditions are approached, the clinically feasible specimen, assay threshold, geographic reproducibility, and safety of manipulating the pathway remain uncertain [21,22,23,27].

Finally, biomarker-development methods remain incomplete. Most analyses use host measurements to explain a microbial association rather than test whether host and microbial features improve prediction together. Few studies use locked external validation, calibration, decision-curve analysis, or subgroup evaluation by histology and treatment. Representation of adenocarcinoma remains limited, and clinically important ecological exposures are recorded inconsistently. Consequently, the current evidence supports biological plausibility but not routine microbiome-guided treatment selection.

### 4.2. Clinical Implementation and Reproducibility

Clinical implementation is limited by substantial pre-analytical, analytical, and biological variability. Differences in sample collection, transport, storage, DNA extraction, sequencing platform, and bioinformatic processing can materially alter measured microbial profiles [32,33]. Low-biomass tumor samples additionally require rigorous negative controls and contamination-aware analysis. Antibiotic exposure is particularly important because it may affect both microbiome composition and response to PD-1 blockade [12]. Diet, geography, smoking, oral health, and concomitant medications should also be recorded prospectively and incorporated into analytical models. Before clinical use, candidate assays will require standardized operating procedures, predefined quality-control criteria and thresholds, acceptable turnaround time and cost, and reproducibility across laboratories and patient populations. Regulatory approval will further require evidence of analytical validity, clinical validity, and clinical utility. These requirements have not yet been satisfied by any microbiome biomarker evaluated in esophageal cancer.

### 4.3. Machine Learning and Predictive Model Validation

Machine-learning methods may facilitate integration of microbial, immune, molecular, and clinical data, but current esophageal-cancer cohorts are generally small relative to the number of candidate features. This high-dimensional setting creates substantial risks of overfitting, data leakage, unstable feature selection, and optimistic performance estimates. Feature selection and model tuning should therefore be restricted to the training data and evaluated using nested cross-validation, followed by locked external validation in an independent cohort. Models should also address missing data, batch effects, histological heterogeneity, and geographic population shifts.

Clinical usefulness cannot be established by discrimination alone. Future studies should report calibration, uncertainty, and decision-curve analysis and should assess performance within clinically relevant subgroups. Interpretability is also important because opaque models may reproduce technical or histological confounding rather than biologically meaningful host-microbiome relationships. Most importantly, microbial features should be compared with a prespecified reference model containing established clinical and host biomarkers. Only a reproducible improvement beyond this benchmark would qualify as clinical predictive integration. Transparent reporting in accordance with TRIPOD+AI guidance will be essential for evaluating these models [34].

## 5. Future Perspective

Future research should follow a staged progression from ecological association to functional characterization, mechanistic validation, and clinical prediction. Multicenter longitudinal studies should include both ESCC and esophageal adenocarcinoma and collect matched saliva, tumor, adjacent tissue, stool, and blood before and during treatment. Strain-resolved metagenomics, microbial transcriptomics, metabolomics, and spatial host-immune profiling should be performed in the same patients. Histology-stratified analyses and systematic documentation of antibiotics, diet, oral health, smoking, and geography will also be essential. Standardized collection, processing, sequencing, and bioinformatic procedures should be adopted to improve reproducibility across centers [32,33].

Candidate microbial genes and metabolites should then undergo causal testing through microbial genetic manipulation, metabolite depletion and rescue, host-pathway perturbation, controlled-colonization models, and patient-derived systems [29]. Only candidates supported by this evidence should advance to predictive modeling. Such models must demonstrate improvement beyond a prespecified clinical-plus-host benchmark and undergo locked external validation, calibration, and clinical-utility assessment, with transparent reporting consistent with TRIPOD+AI guidance [34]. Microbiome-directed interventions should proceed to clinical testing only after their mechanism, safety, and treatment-specific effects have been established.

**Figure 2:**
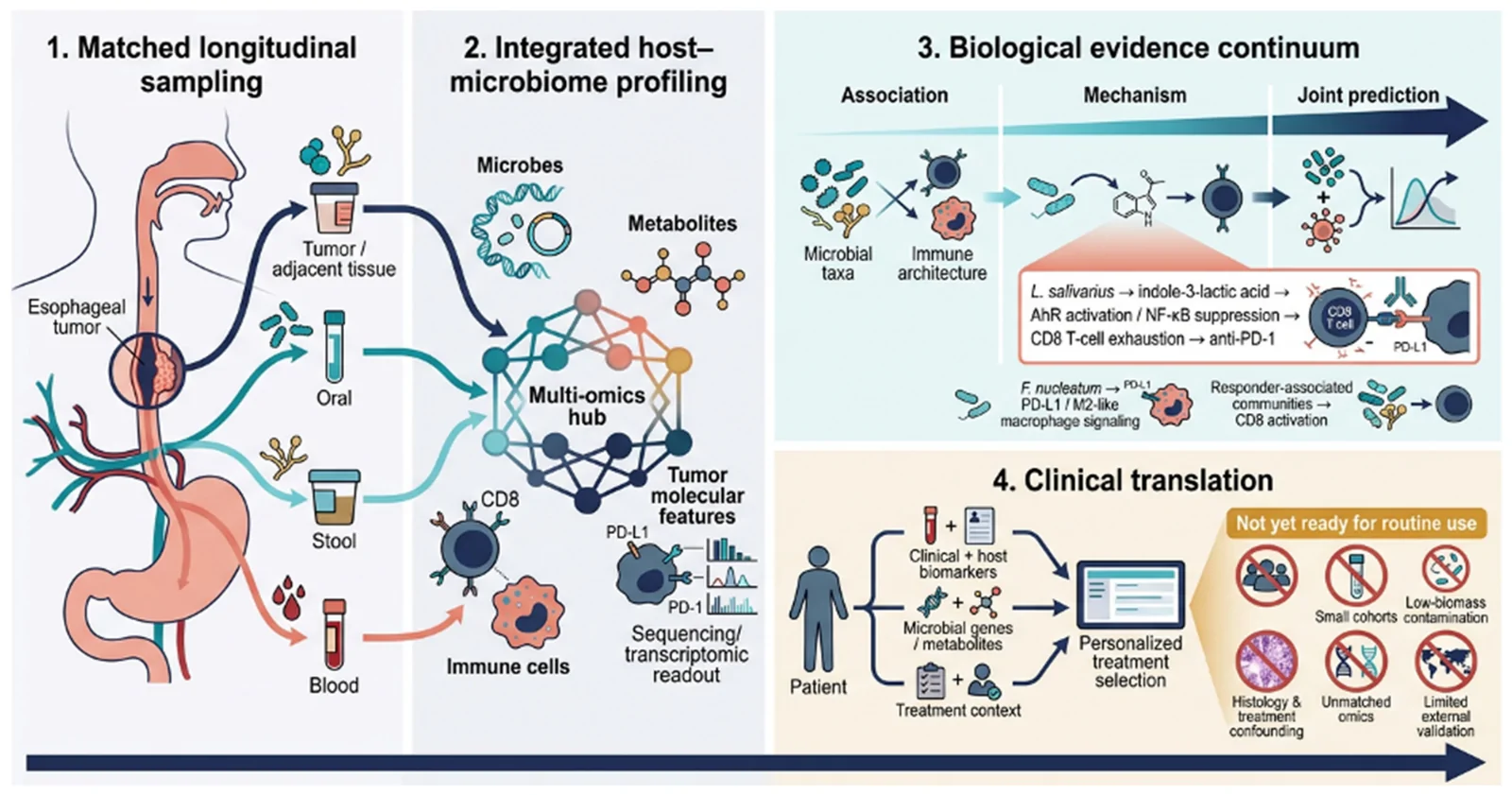
An Envisioned Framework for Future Host-Microbiome Research in Esophageal Cancer: This figure illustrates an envisioned framework for future host-microbiome research in esophageal cancer, integrating longitudinal multi-compartment sampling, multi-omic profiling, mechanistic validation, and externally validated prediction toward personalized treatment selection.

Encouragingly, these recommendations are beginning to be operationalized. The prospective PKU-ESCC-Monitor study (NCT07152535) plans to characterize the dynamic evolution of ESCC using integrated multi-omics (tissue genomics, circulating tumor DNA, imaging, immune profiling, and the microbiome) across both a perioperative cohort receiving neoadjuvant therapy and an advanced cohort receiving first-line immunotherapy [35]. Such efforts illustrate the feasibility of the matched, longitudinal, multi-layer sampling advocated here, though their value for treatment-response prediction will ultimately depend on the nested, externally validated modeling framework outlined above. Figure 2 represents a envisioned framework for future research project. 

## 6. Conclusions

Current evidence supports biologically meaningful host-microbiome interactions in esophageal cancer, but the strength of evidence varies substantially. Most studies demonstrate ecological or functional associations, whereas only a limited number establish a microbial factor-host pathway-immune consequence chain [22,28]. No study has yet demonstrated clinical predictive integration through externally validated improvement beyond established clinical and host biomarkers. Future progress will therefore require functionally resolved measurements, matched biological compartments, histology-specific analyses, standardized methods, stronger causal experiments, and rigorous predictive validation. Overall, biological integration remains considerably more advanced than clinical prediction, and microbiome-guided treatment selection should remain investigational until this gap is addressed.

## Figures and Tables

**Table 1:** Evidence hierarchy, validation status, and translational readiness of studies integrating microbial and host-associated measurements in esophageal cancer.

Study	Clinical Context	Integrated Microbial-Host Finding	Evidence Level	Validation Status	Clinical Readiness
Zhang et al. (2023) [18]	98 resected ESCCs; tumor tissue	Greater intratumoral bacterial diversity and higher *Lactobacillus* abundance were associated with fewer NK cells, more PD-L1-positive epithelial cells and TAMs, and poorer overall survival.	L1: Ecological association	Single observational cohort; PCR/FISH supported microbial localization; no external or functional validation.	Discovery
Kosumi et al. (2023) [19]	300 resected esophageal cancers; tumor tissue	Intratumoral *F. nucleatum* was associated mainly with a weaker peritumoral lymphocytic reaction, but not with global T-cell densities or checkpoint-marker expression.	L1: Ecological association	Large observational cohort; targeted microbial quantification; no functional or external predictive validation.	Discovery
Wang et al. (2025) [20]	213 ESCCs; tumor and adjacent tissue	*Bifidobacterium* positivity was associated with lower FOXP3-positive or global TIL measures and with compartment-dependent nutritional and skeletal-muscle indices, but not overall survival.	L1: Ecological association	Single cohort with paired anatomical compartments; no causal or external validation.	Discovery
Liang et al. (2025) [21]	105 ESCCs with an independent test cohort; pretreatment stool	Responder-enriched fungal communities were associated with Th1 cytokines, and *Candida boidinii* enhanced CD8 infiltration and anti-PD-1 activity in mice. The independently tested classifiers used fungal features alone rather than a joint host-mycobiome model.	L1: Ecological association	Independent test cohort for the fungal-only classifier plus experimental mouse support; no defined fungal effector-host molecular pathway or externally validated joint model.	Biologically supported
Zhou et al. (2026) [22]	43-patient discovery cohort plus validation cohorts; longitudinal stool	*L. salivarius*-derived indole-3-lactic acid activated AhR, suppressed NF-κB signaling, promoted terminal exhaustion of NKG7-positive CD8 progenitor-exhausted T cells, and induced anti-PD-1 resistance. A joint model incorporated *L. salivarius*, PD-L1, and T-cell-state measurements.	L3: Mechanistic integration	Human validation cohorts supported the microbial signal; bacterial genetic disruption, metabolite rescue, and host-pathway perturbation supported causality. The joint model was not evaluated through locked external validation, calibration, or incremental comparison with a prespecified clinical-plus-host benchmark.	Candidate predictive model
Wu et al. (2023) [23]	25 ESCCs receiving neoadjuvant chemoimmunotherapy; tumor microbiota	Responder-enriched *Streptococcus* was associated with CD8-positive and granzyme B-positive infiltration and longer disease-free survival. Responder-derived microbiota or *Streptococcus* improved anti-PD-1 activity in mice, and the benefit was lost after CD8 depletion.	L1: Ecological association	Experimental colonization and CD8-depletion studies supported biological activity; no defined microbial effector–host molecular pathway or external patient-level joint classifier.	Biologically supported
Shaikh et al. (2024) [25]	23 operable esophageal/GEJ cancers, predominantly adenocarcinoma; stool	Responder-enriched taxa were associated with fecal C16 ceramide and chenodeoxycholic acid and with pathological complete response. Microbial origin of the metabolites and a direct host molecular target were not established.	L2: Functional association, qualified	Exploratory cohort with only eight pathological complete responses; no external validation, metabolite-source confirmation, or host-target validation.	Discovery
Zhang et al. (2025) [27]	Multiple small ESCC tissue subsets	*F. nucleatum*-associated phenyllactic acid increased CLEC12A expression and favored M2-like macrophage polarization; CLEC12A knockdown attenuated macrophage polarization and IL-4/IL-10 production.	L2: Functional association	In vitro functional experiments and host-target knockdown supported pathway discovery, but patient multi-omic layers were not fully matched and microbial metabolite origin was not definitively established.	Biologically supported
Liu et al. (2025) [30]	TCGA esophageal-cancer cohort	Microbiome-associated molecular subtypes differed in transcriptomic, methylation, mutational, immune, and computational response features, but separation was strongly confounded by adenocarcinoma versus squamous histology.	L1: Computational ecological association	Internal computational integration; no external clinical validation, observed treatment-response validation, or demonstration of microbial causality.	Discovery
Li et al. (2023) [28]	ESCC patients receiving immunotherapy; tumor tissue and serum, with mechanistic validation	Intratumoral *F. nucleatum* and serum anti-*F. nucleatum* IgG were associated with nonresponse. The Fn-Dps virulence factor promoted ATF3-dependent PD-L1 upregulation and impaired T-cell proliferation and cytokine secretion.	L3: Mechanistic integration	Experimental validation supported the Fn-Dps-ATF3-PD-L1 pathway; no prospective externally validated joint microbial-host predictive model.	Biologically supported
He et al. (2024) [24]	79 initially inoperable LAESCCs before chemoradiotherapy; pCR assessed in 26 surgical patients	Baseline salivary microbial features were associated with pathological complete response, peripheral CD3/CD8 T-cell changes, and serum cytokines, but microbial and host features were not jointly modeled.	L1: Ecological association	Exploratory response subset; no independent external validation or integrated microbial-host model	Discovery

Evidence hierarchy: L1, ecological association: microbial composition, diversity, or taxa associated with host phenotype or outcome; L2, functional association: microbial genes, pathways, or metabolites associated with host biology without a complete causal chain; L3, mechanistic integration: a defined microbial factor experimentally linked to a host molecular pathway and immune or treatment consequence. Clinical-readiness categories: discovery; biologically supported; candidate predictive model; clinically validated. No reviewed study met L4 criteria or achieved clinically validated readiness. Abbreviations: AhR: aryl hydrocarbon receptor; ESCC: esophageal squamous cell carcinoma; FISH: fluorescence in situ hybridization; GEJ: gastroesophageal junction; NK: natural killer; PD-L1: programmed death-ligand 1; TAM: tumor-associated macrophage; TIL: tumor-infiltrating lymphocyte.

## Data Availability

No new data were created or analyzed in this study. Data sharing is not applicable to this article.
